# Evolution of high tooth replacement rates in theropod dinosaurs

**DOI:** 10.1371/journal.pone.0224734

**Published:** 2019-11-27

**Authors:** Michael D. D’Emic, Patrick M. O’Connor, Thomas R. Pascucci, Joanna N. Gavras, Elizabeth Mardakhayava, Eric K. Lund

**Affiliations:** 1 Department of Biology, Adelphi University, Garden City, New York, United States of America; 2 Department of Anatomical Sciences, Stony Brook University, Stony Brook, New York, United States of America; 3 Department of Biomedical Sciences, Ohio University, Athens, Ohio, United States of America; 4 Ohio Center for Ecology and Evolutionary Studies, Athens, Ohio, United States of America; 5 Department of Biological Sciences, North Carolina Museum of Natural Sciences, Raleigh, North Carolina, United States of America; Monash University, AUSTRALIA

## Abstract

Tooth replacement rate is an important contributor to feeding ecology for polyphyodont animals. Dinosaurs exhibit a wide range of tooth replacement rates, mirroring their diverse craniofacial specializations, but little is known about broad-scale allometric or evolutionary patterns within the group. In the current broad but sparse dinosaurian sample, only three non-avian theropod tooth replacement rates have been estimated. We estimated tooth formation and replacement rates in three additional non-avian theropod dinosaurs, the derived latest Cretaceous abelisaurid *Majungasaurus* and the more generalized Late Jurassic *Allosaurus* and *Ceratosaurus*. We created the largest dental histological and CT dataset for any theropod dinosaur, sectioning and scanning over a dozen toothed elements of *Majungasaurus* and several additional elements from the other two genera. Using this large sample, we created models of tooth formation time that allow for theropod replacement rates to be estimated non-destructively. In contrast to previous results for theropods, we found high tooth replacement rates in all three genera, with *Allosaurus* and *Ceratosaurus* rates of ~100 days and 56 days for *Majungasaurus*. The latter rate is on par with those of derived herbivorous dinosaurs including some neosauropods, hadrosaurids, and ceratopsians. This elevated rate may be a response to high rates of tooth wear in *Majungasaurus*. Within Dinosauria, there is no relationship between body mass and tooth replacement rate and no trends in replacement rate over time. Rather, tooth replacement rate is clade-specific, with elevated rates in abelisaurids and diplodocoids and lower rates in coelurosaurs.

## Introduction

Tooth formation time and replacement rate are related to feeding mechanics and diet and can help reconstruct paleoecology and paleodemography [1–3]. Controls on tooth formation and replacement rates in relation to body mass, feeding ecology, and phylogeny, especially regarding the loss of teeth on the lineage leading to birds, remain debated [4,5]. Tooth formation and replacement rates have been measured empirically in a variety of extant taxa; in these groups, counts of incremental lines of von Ebner within dentine of successive teeth in a tooth socket can be used to estimate such rates ([1,6]. Based on data from nine species, Erickson [1] found that both tooth formation time in Dinosauria, and tooth replacement rate in Theropoda, were negatively correlated with tooth volume, suggesting that structural or physiological limits on odontoblast activity constrained the rate of tooth growth regardless of tooth size or body mass. Erickson [1] also estimated that the three carnivorous (theropod) dinosaurs in his sample had tooth replacement rates on the order of several months to years, whereas the herbivorous dinosaurs had tooth replacement rates on the order of 2–3 months.

An explosion in the number of described dinosaur taxa over the last two decades has expanded the range of dental and craniofacial morphologies known in the clade, prompting reevaluation of hypotheses explaining tooth formation and replacement times with a larger sample size and increased taxonomic breadth. To do this, we established a rich histological and tomographic dental and craniofacial dataset of the carnivorous dinosaur *Majungasaurus crenatissimus*, which has an apomorphic anteroposteriorly short and dorsoventrally tall skull with abbreviated tooth crowns relative to most other theropods ([7]. This is the largest such dataset for a Mesozoic dinosaur, allowing us to also investigate allometric and intraspecific variation within *Majungasaurus*. We also collected similar but smaller datasets for the non-avian theropods *Allosaurus* and *Ceratosaurus* to both contextualize our findings in *Majungasaurus* and better examine phylogenetic and allometric trends and possible constraints on odontogenesis across Dinosauria. By examining these trends, we test the hypothesis that carnivorous dinosaurs had lower tooth replacement rates than herbivorous ones as found by Erickson [1].

## Methods

We CT scanned 52 isolated *Majungasaurus* teeth and 15 *Majungasaurus* tooth-bearing elements at Stony Brook University Hospital that are accessioned at the Field Museum of Natural History, Chicago, USA (FMNH), Université d’Antananarivo, Antananarivo, Madagascar (UA), Stony Brook University (MAD, Madagascar Collection), or Denver Museum of Nature and Science (DMNH) (see S1 Fig and Morphobank project P322 or Morphosource project 848 for specimen numbers). We scanned 11 *Allosaurus* (Museum of Paleontology, Brigham Young University, Provo, Utah (BYU), BYU 8901, 2028) and one *Ceratosaurus* (BYU 12893) tooth-bearing element(s) at Orem Community Hospital. We measured 104, 32, and 6 apicobasal tooth lengths based on these CT data for *Majungasaurus*, *Allosaurus*, and *Ceratosaurus*, respectively. Linear measurements of the *Majungasaurus* teeth were taken according to the approach utilized in Smith [8]; volumes of isolated teeth were measured by water displacement (S1 File).

We thin sectioned 19 of the CT scanned *Majungasaurus* teeth, one *Ceratosaurus* tooth (Museum of Western Colorado, Grand Junction, Colorado (MWC), MWC 1), and one *Allosaurus* tooth (BYU 2028) according to standard paleohistological techniques [9]. Specimens were embedded in epoxy resin, bisected in the mesiodistal plane, ground with 600 grit sandpaper, mounted to glass slides with cyanoacrylate, cut to ca. 0.5 mm, and sanded again using 600 and then 1200 grit sandpaper to a thickness of ca. 100 *μm*. Poor preservation of incremental lines left us able to only measure incremental line thicknesses in 13 *Majungasaurus* teeth and estimate tooth age in only five. Thin sections were stitched at 5x or 20x using a Zeiss Axioimager Z2 system running Zen2 software. We measured incremental line thicknesses and counted incremental lines on these stitched images to obtain individual tooth formation times (interpolating for lines that were diagenetically altered; see Morphobank Project 3222, Morphosource Project 848 and S1 File).

Some studies [10,11] have questioned previous interpretation of incremental lines of von Ebner in dinosaurs, instead suggesting that they represent higher-order increments with implied absence of daily lines due to preservation issues. We refute this hypothesis with two lines of evidence: first, the ca. 10–20 *μm* mean thicknesses of the dinosaurian daily incremental lines is on par with those found via labeling studies of extant archosaurs [6,12]; second, if these were higher order increments such as Andresen lines, they would imply tooth formation and replacement times 7–14 times longer than those previously estimated based on body mass [13]. These augmented times would be biologically unreasonable (e.g., 3.5–7 years to form a single *Diplodocus* tooth or 15–30 years to replace a single *T*. *rex* tooth). Therefore, we continue to interpret ca. 10–20 *μm* thick increments in dentine as daily incremental lines of von Ebner in archosaurs.

For *Majungasaurus*, we regressed total tooth length (measured parallel to the distal margin) versus tooth formation time to allow for prediction of formation time in teeth that could not be thin sectioned. We used PAST3 [14] to compare the fit of nine regression models to *Majungasaurus* data collected herein using Akaike’s information criterion corrected for small sample size (AICc; see S1 File for values). We also fit these nine models and compared AICc scores for previously published *Diplodocus* and *Camarasaurus* data [2]. A power model fit the data best for *Majungasaurus*, a Gaussian model fit the data best for *Camarasaurus*, and both quadratic and Logistic models fit equally well for *Diplodocus* (AICc values only differed by ~1). We follow D’Emic et al. [2] in using a quadratic model to describe tooth age-length relationships on theoretical grounds: tooth growth should be rapid in the apicobasal direction at first, as odontoblast apposition is more parallel to the direction of tooth extension, and then slow as the direction of apposition becomes more perpendicular to the overall direction of tooth growth. Furthermore, the *Camarasaurus* and *Majungasaurus* data overlap (see Results below), suggesting that a single model should describe their growth–length relationship. However, AICc values favor different models of tooth growth, which is more likely a statistical artifact related to the limited sample sizes involved. The quadratic *Majungasaurus* regression model was used to estimate tooth age based on the CT data of dentigerous elements for *Majungasaurus*, *Ceratosaurus*, and *Allosaurus*, justified by the similar proportions of these teeth.

Tooth formation time, replacement rate, and incremental line thicknesses for other dinosaurian taxa were gathered from the literature [1,2,15,16]. We plotted tooth replacement rates on a phylogeny with branch lengths compiled from the literature [17]; only adult values were used because tooth replacement rates change over ontogeny [1,6]. Branch lengths were based on specimen geologic ages and assigned using the R code provided by Graeme Lloyd (http://www.graemetlloyd.com/methdpf.html). No geologic age was available for the titanosaur premaxilla included in the dataset; for our analysis it was estimated as 83 Ma based on its reported “Upper Cretaceous” provenance [18]. This specimen was omitted from regressions because it was not possible to estimate its body mass, so sensitivity analysis surrounding its geologic age was unnecessary. We regressed incremental line width and then tooth replacement rate on ln(body mass), both with and without accounting for phylogenetic influence following the methods and using the R code provided in D’Emic et al. [19]. The R code, raw data, phylogenetic tree, and branch lengths used herein are provided in S2–S6 Files. Again, only adult values were used to avoid confounding ontogenetic factors. Body masses were estimated using stylopodial limb circumferences and the equations presented in Campione and Evans [20] and Campione et al. [21]; circumferences were sourced from Benson et al. [17] or personally measured by the first author. Because a humeral circumference is not available for *Patagosaurus*, we estimated its humeral circumference as 578 mm based on the ratio in the closely related *Cetiosaurus oxoniensis*, yielding an estimated body mass of 27,221 kg. We assessed the sensitivity of our regressions to this coarse estimate by re-running the analysis with 50% larger and smaller values for *Patagosaurus* (i.e., using 13,612 kg or 40,832 kg as its body mass estimate).

## Results

### New theropod data

The total volume of the sectioned *Ceratosaurus* tooth (MWC 1, a posterior maxillary or dentary tooth) is 1.1 ml, its preserved length is 40 mm, and its mesiodistal dimension is 13 mm. The *Allosaurus* tooth was incomplete and so these measurements are not available. The crown height and mesiodistal length of a mid-dentary crown of *Majungasaurus* are 26 and 13.5 mm, respectively [8]. We take this as an approximate mean size for the functional dentition following Erickson [1,6]. Isolated *Majungasaurus* teeth similar in size to this have volumes of about 3 ml as measured by water displacement (Electronic Supplementary Material), which we use for our mean *Majungasaurus* tooth volume.

**Fig 1:**
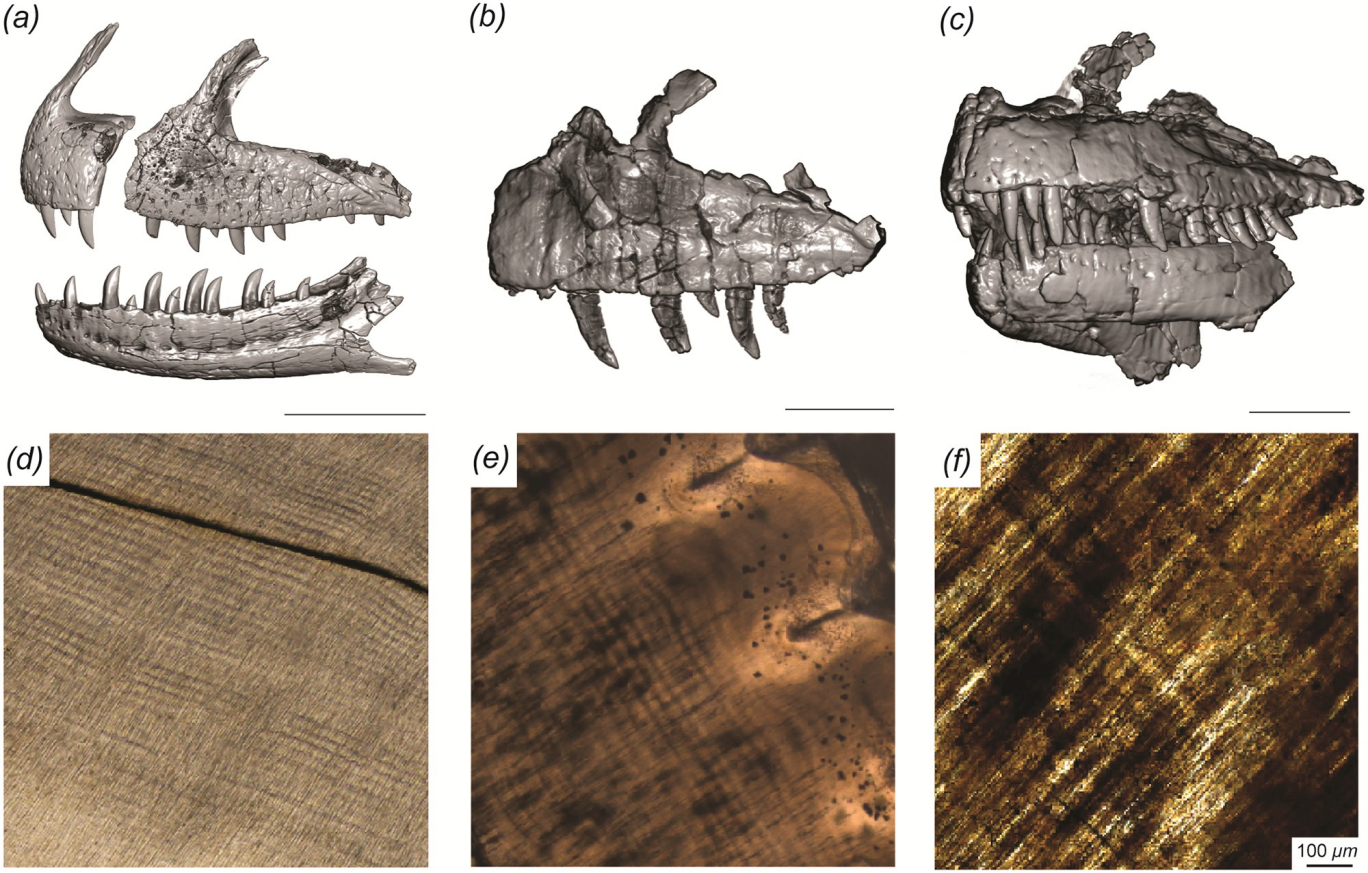
Craniofacial and dental histology of the theropod dinosaurs included in this study. (A) *Allosaurus* (BYU 8901), (B) *Ceratosaurus* (BYU 12893) and (C) *Majungasaurus* (FMNH PR 2278) surface reconstructions derived from computed tomography data and dentine histology. Scale bars below each cranial element(s) equal 10 cm. Histological sections derived from (D) *Majungasaurus* (MAD 07757), (E) *Ceratosaurus* (MWC 1), and (F) *Allosaurus* (BYU 2028), illustrating incremental daily lines (von Ebner) in dentine, which extend obliquely from upper left to lower right in each image. Scale bar of 100 *μm* applies to (D–F).

*Majungasaurus* has incremental line thicknesses (mean and standard deviation: 18 +/- 5 *μm*) within the range of those reported by Erickson [1] and D’Emic et al. [2] for other archosaurs, whereas those of *Allosaurus* were slightly thicker (23 +/-1 *μm*) and those of *Ceratosaurus* were slightly thinner (14 +/- 3 *μm*; Fig 1). The largest estimated tooth formation times for these three genera are estimated as 293 (*Majungasaurus*), 359 (*Allosaurus*), and 337 days (*Ceratosaurus*), which is on par with tooth formation times in sauropod dinosaurs with similar tooth size [2]. CT data reveal that successive generations of replacement teeth are more similar in size in *Majungasaurus* than in *Allosaurus* or *Ceratosaurus*. Using the length-tooth age model developed below to predict tooth age from tooth length measured in CT slices, replacement rates for these three genera are estimated at 56, 104, and 107 days, respectively.

### Models of tooth formation time

**Fig 2:**
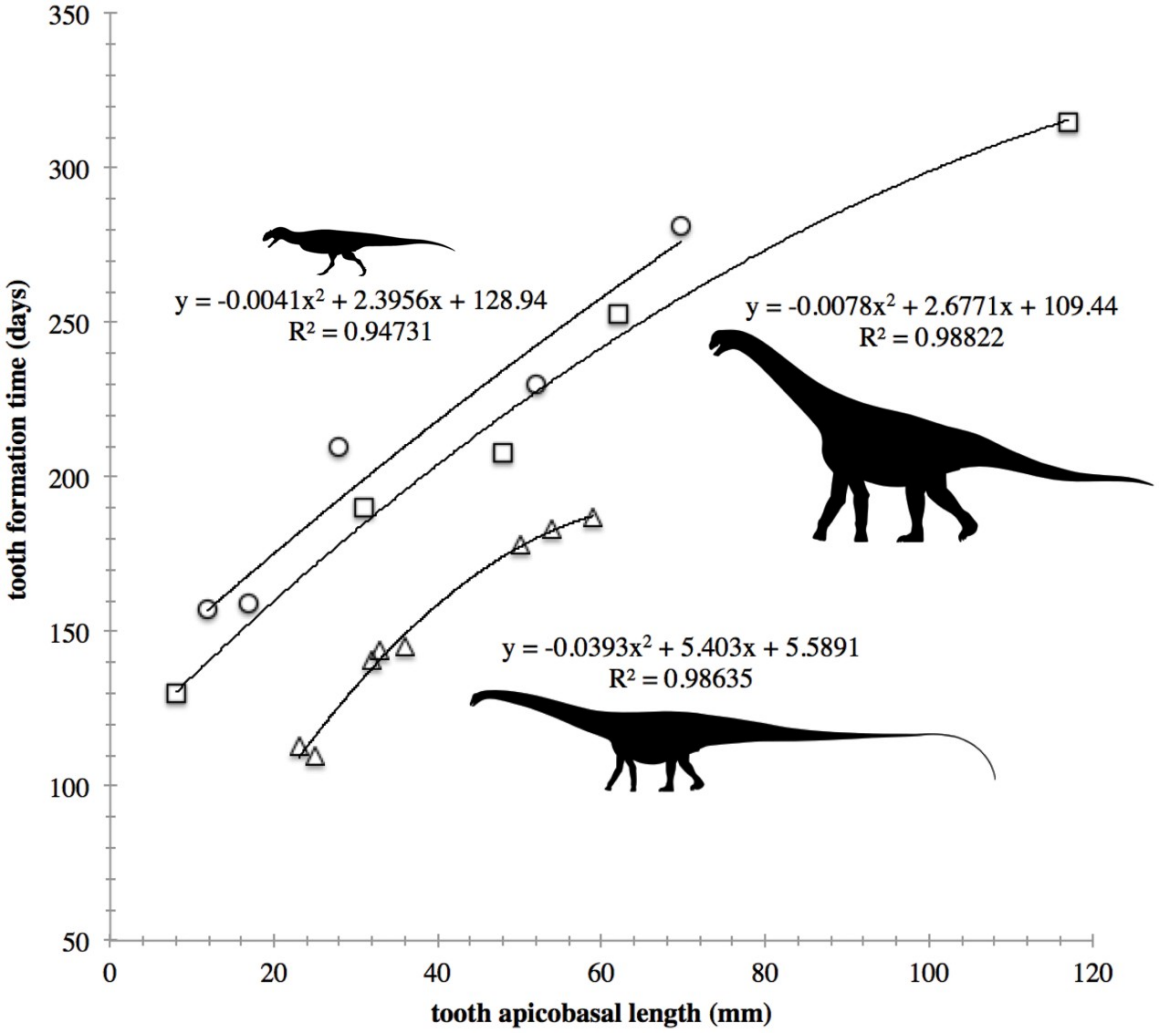
Models of tooth formation time. Tooth Formation time versus tooth apicobasal length for *Diplodocus* (triangles), *Camarasaurus* (squares), and *Majungasaurus* (circles). Sauropod data from [2]. Silhouettes by S. Hartman from www.phylopic.org.

Thin sections of *Majungasaurus* teeth reveal a tight relationship between tooth apicobasal length and tooth age as measured by counting daily deposited incremental lines of von Ebner (Fig 2; r^2^ = 0.95; *p* = 0.05). Interestingly, the relationship is nearly identical to that obtained for *Camarasaurus* [2]; however, it is substantially different than the relationship for *Diplodocus* (Fig 2). This is likely because *Majungasaurus* and *Camarasaurus* have teeth of similar aspect ratio (slenderness index [SI] = crown length:crown mesiodistal length ca. 1.9; Electronic Supplementary Material; [22]), whereas *Diplodocus* has much narrower crowns (SI = 5.2; [22]).

### Allometric and Phylogenetic patterns

**Fig 3:**
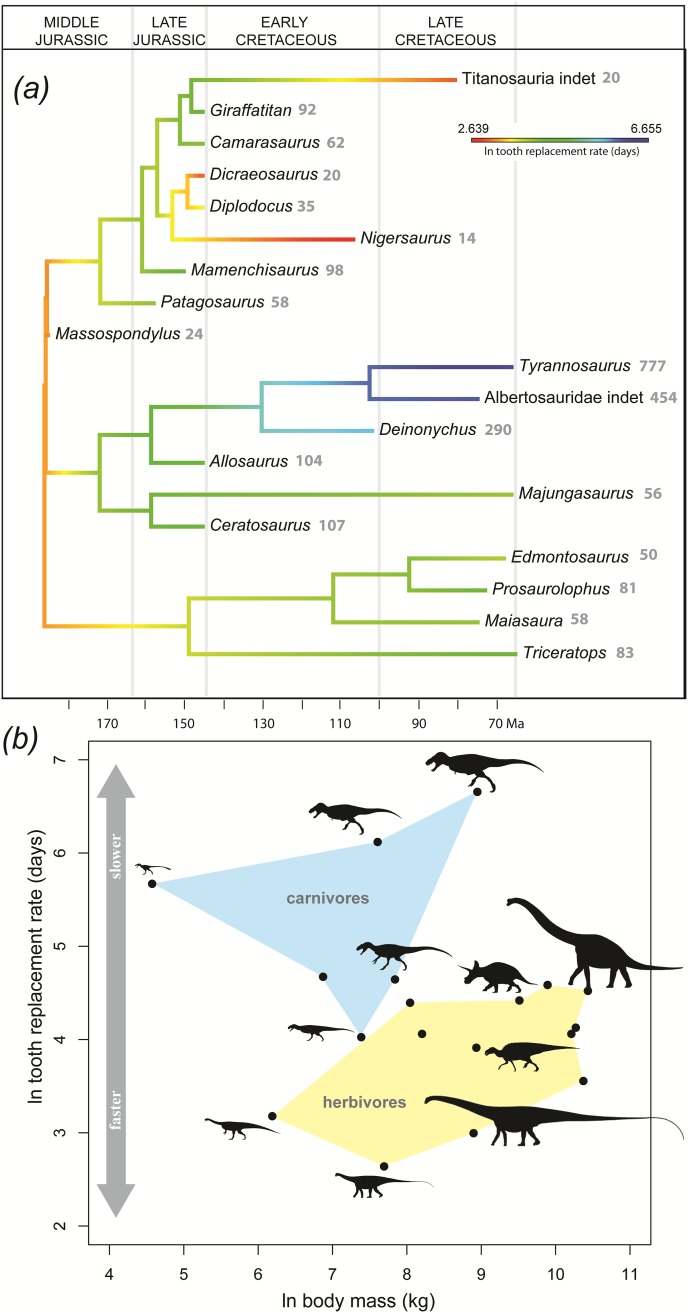
Phylogeny, time, and body mass in relation to tooth replacement rate. (A) Natural log of tooth replacement rates plotted on a time-calibrated phylogeny [17] of dinosaurs. Grey numbers indicate tooth replacement rates in days. (B) Natural log of body mass versus tooth replacement rate in dinosaurs. Blue polygon indicates carnivorous taxa, yellow polygon indicates herbivorous taxa. Data for *Majungasaurus*, *Ceratosaurus*, and *Allosaurus* are from this paper; data from *Dicraeosaurus* are from Schwarz et al. [16]; data from *Giraffatitan* are from Kosch et al. [15]; data from other sauropodomorphs are from D’Emic et al. [2]; data for other taxa are from Erickson [1]. ‘Titanosauria indet.” refers to the titanosaur premaxilla described by Coria and Chiappe [18] and analyzed by D’Emic et al. [2]. Silhouettes by S. Hartman and M. Wedel from www.phylopic.org.

In dinosaurs, incremental line width is unrelated to body mass (OLS regression: r^2^ = 0.28; *p* = 0.07), as is tooth formation time (r^2^ = 0.008; *p* = 0.72). No relationship between body mass and tooth replacement rate was detected whether or not phylogenetic information was included in the regression (Fig 3; OLS regression: r^2^ = 0.024; *p* = 0.54; PGLS regression: lambda ~ 1). This relationship was still not significant when only herbivores were included in the regression (OLS: r^2^ = 0.53; *p* = 0.07). Uncertainly surrounding our body mass estimate for *Patagosaurus* is immaterial to the results: increasing or decreasing its body mass estimate by 50% does not meaningfully alter the significance, strength (i.e., r value) or direction of any of our regression results.

We infer approximately monthly tooth replacement rates as the ancestral dinosaurian condition (Fig 3B), though this should be viewed as a preliminary estimate until better sampling is conducted near the base of the tree. Coelurosaurs and diplodocoids have unusually slow and fast replacement rates, respectively (Fig 3). Even excluding these two groups, there is no relationship between body mass and replacement rate (r^2^ = 0.12; *p* = 0.27). Carnivores have significantly lower absolute tooth replacement rates than do herbivores (t test: *p* < 0.001; Mann-Whitney U test: *p* = 0.001). In dinosaurs, feeding ecology is a better predictor of tooth replacement rate than is body mass.

## Discussion and conclusions

We established a novel dataset to address relationships among tooth formation time, tooth replacement rate, and body mass in an expanded dinosaurian sample, including the largest sample of individuals for any single dinosaur species (*Majungasaurus*). This allowed us to develop tooth age-length models in an aim to estimate tooth formation and replacement rates in a non-destructive way. In the future, novel CT data for additional dinosaur species can be used with our models to estimate tooth formation and replacement rates, refining when, where, and in precisely which clades elevated tooth replacement rates evolved. Increased histological sampling is necessary to test that the tooth age-length models developed for *Camarasaurus*, *Majungasaurus*, and *Diplodocus* are valid for a wide variety of dinosaurs with teeth of similar aspect ratio to those of these genera.

Our expanded sample supports the finding of Erickson [1] that structural or physiological limitations on dentine production rate by odontoblasts affect both tooth generation and replacement rates. Incremental line thickness does not increase with body mass, instead hovering around a relatively consistent ~10–20 *μm*. This further supports the concept that there may indeed be a biological limit on odontogenesis that requires other evolutionary innovations to overcome increased selection pressures imposed by faster rates of tooth wear, such as the development of tooth batteries in some ornithischian and sauropod dinosaurs [1,23] or the evolution of ever-growing teeth as in some groups of mammals [24].

All carnivorous dinosaurs in our sample replaced their teeth more slowly than the herbivorous dinosaurs with the exception of *Majungasaurus*, which has tooth replacement rates comparable to those of broad-toothed sauropods and dental-battery bearing ceratopsians and hadrosaurids. Tooth replacement rate is intrinsically controlled at the individual level and does not respond to increased wear or breakage [25]; instead, tooth replacement rate is a general reflection of dietary specialization [1,3,6]. The generally slower replacement rate for theropods versus herbivores likely reflects their softer foodstuffs and/or release from the necessity of fast tooth replacement via adaptations against tooth breakage or rapid wear [3]. In contrast, the elevated, herbivore-like rate for the carnivorous *Majungasaurus* perhaps reflects its osteophageous behavior, evidenced by extensive tooth markings on both herbivores and conspecifics in the Maevarano Formation [26]. We hypothesize that elevated tooth replacement rates characterize at least some other abelisaurids, which have maxillae “packed with replacement teeth” (Sereno and Brusatte [27]:20); by extension, perhaps osteophageous behavior also applies to these species. *Tyrannosaurus rex* likely exhibited osteophageous behavior, but likely took a different evolutionary approach compared to *Majungasaurus* and perhaps other abelisaurids, evolving exceedingly robust teeth and slow replacement rates [1,28]. These disparate approaches to increased consumption of harder foodstuffs in theropods seem to mirror the evolution of broad-crowned, faster replacing, vs. narrow-crowned, slower-replacing teeth in sauropods [1].

## Supporting information

S1 Fig Labeled image of surface models showing specimen numbers for CT scanned isolated *Majungasaurus crenatissimus* teeth. (TIF)

S1 File Excel spreadsheet with gross tooth measurements for *Majungasaurus*, dinosaur incremental line thicknesses, input data for the tooth age-length model, estimated tooth formation times and replacement rates, and regression data. (XLSX)

S2 File R code to perform analyses. (R)

S3 File ln(tooth replacement rates) in several dinosaurs. (CSV)

S4 File Branch lengths (in millions of years) for calibrating phylogeny. (TXT)

S5 File ln(body mass) and ln(tooth replacement rates) in several dinosaurs (.txt file) for regression. (TXT)

S6 File Phylogenetic tree (.nex file) for use in R code. (NEX)
