# Correction: Evolution of high tooth replacement rates in theropod dinosaurs

**DOI:** 10.1371/journal.pone.0226897

**Published:** 2019-12-26

**Authors:** Michael D. D’Emic, Patrick M. O’Connor, Thomas R. Pascucci, Joanna N. Gavras, Elizabeth Mardakhayava, Eric K. Lund

There are errors in the caption for Fig 1. Please see the correct Fig 1 caption here.

**Fig 1:**
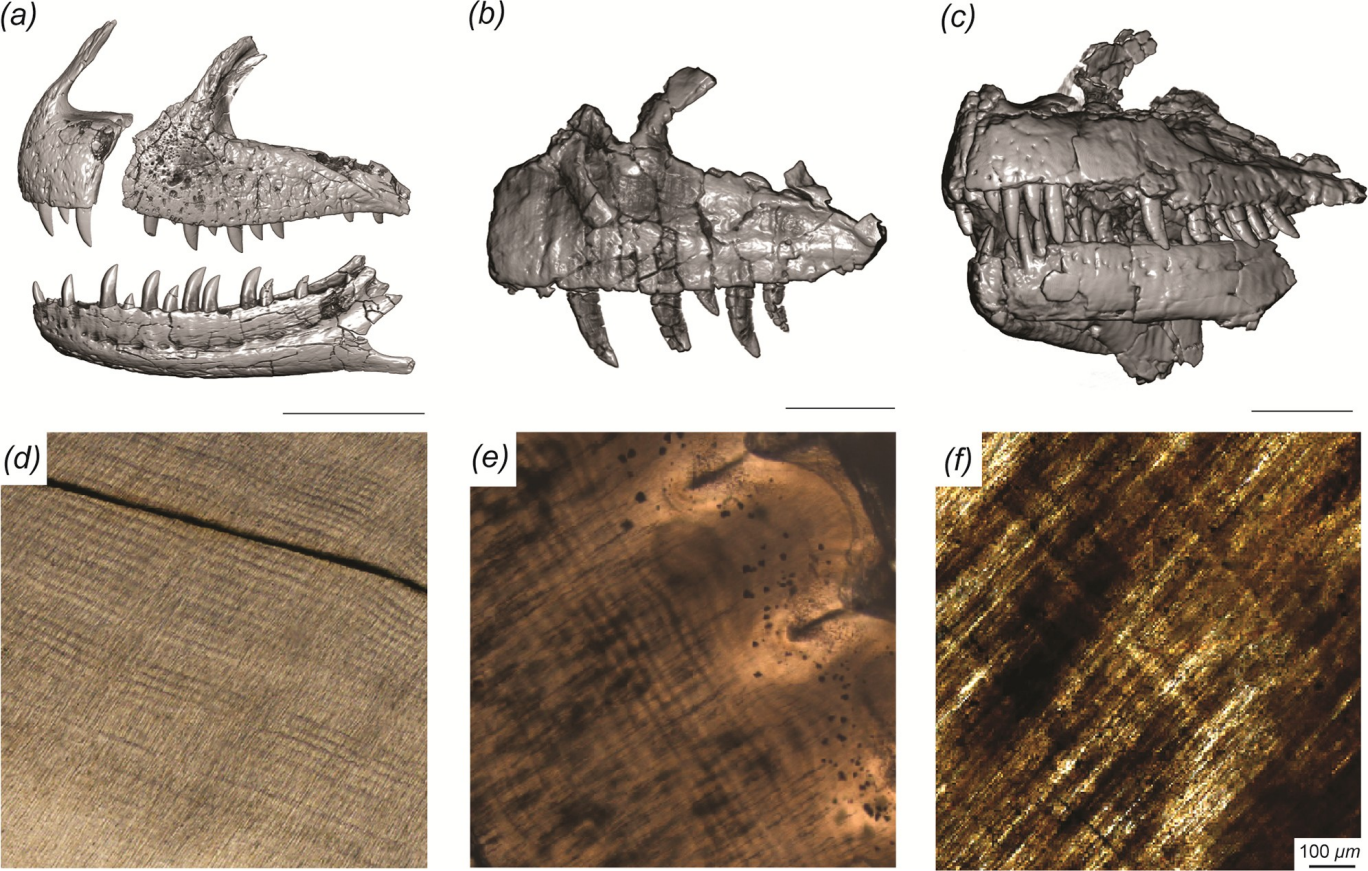
Craniofacial and dental histology of the theropod dinosaurs included in this study. (A) *Majungasaurus* (FMNH PR 2278), (B) *Ceratosaurus* (BYU 12893), and (C) *Allosaurus* (BYU 8901) surface reconstructions derived from computed tomography data and dentine histology. Scale bars below each cranial element(s) equal 10 cm. Histological sections derived from (D) *Majungasaurus* (DMNH EPV.134369), (E) *Ceratosaurus* (MWC 1), and (F) *Allosaurus* (BYU 2028), illustrating incremental daily lines (von Ebner) in dentine, which extend obliquely from upper left to lower right in each image. Scale bar of 100 μm applies to (D–F).

S1 File is missing museum accession numbers. Please view the correct S1 File below.

## Supporting information

S1 File Excel spreadsheet with gross tooth measurements for *Majungasaurus*, dinosaur incremental line thicknesses, input data for the tooth age-length model, estimated tooth formation times and replacement rates, and regression data. (XLSX)

## References

[1] D’Emic MD, O’Connor PM, Pascucci TR, Gavras JN, Mardakhayava E, Lund EK (2019) Evolution of high tooth replacement rates in theropod dinosaurs. PLoS ONE 14(11): e0224734 10.1371/journal.pone.0224734 31774829 PMC6880968

