# Supplementary figures and images for: Evolution of high tooth replacement rates in theropod dinosaurs
Source: PLoS One. 2019 Nov 27;14(11):e0224734. doi: 10.1371/journal.pone.0224734 (PMC6880968; doi:10.1371/journal.pone.0224734)

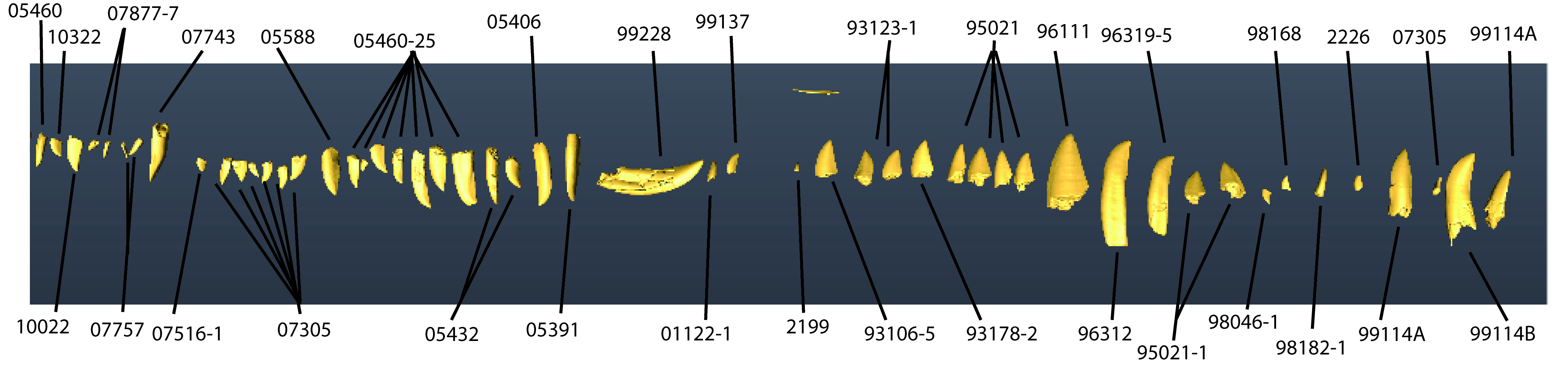

Supplement: S1 Fig — (TIF) [file pone.0224734.s001.tif]
